# *Aeromonas* spp. in Freshwater Bodies: Antimicrobial Resistance and Biofilm Assembly

**DOI:** 10.3390/antibiotics13020166

**Published:** 2024-02-08

**Authors:** Maria Nascimento, Joao Rodrigues, Rui Matias, Luisa Jordao

**Affiliations:** 1 Department of Environmental Health (DSA), National Institute of Health Dr. Ricardo Jorge (INSA), Avenida Padre Cruz, 1649-016 Lisboa, Portugal; maria_d_n@hotmail.com; 2 Department of Infectious Diseases (DDI), National Institute of Health Dr. Ricardo Jorge (INSA), Avenida Padre Cruz, 1649-016 Lisboa, Portugal; joao.rodrigues@insa.min-saude.pt (J.R.); rui.matias@insa.min-saude.pt (R.M.)

**Keywords:** *Aeromonas* spp., biofilm, freshwater, chlorination, antibiotic resistance

## Abstract

*Aeromonas* spp. are environmental bacteria able to infect animals and humans. Here, we aim to evaluate the role of biofilms in *Aeromonas* persistence in freshwater. *Aeromonas* were isolated from water and biofilm samples and identified by Vitek-MS and *16S rRNA* sequencing. Antibiotic susceptibility profiles were determined according to EUCAST, and a crystal violet assay was used to assess biofilm assembly. MTT and the enumeration of colony-forming units were used to evaluate biofilm and planktonic *Aeromonas* susceptibility to chlorination, respectively. Identification at the species level was challenging, suggesting the need to improve the used methodologies. Five different *Aeromonas* species (*A. salmonicida*, *A. hydrophila*, *A. media*, *A. popoffii* and *A. veronii*) were identified from water, and one species was identified from biofilms (*A. veronii*). *A. veronnii* and *A. salmonicida* presented resistance to different antibiotics, whith the highest resistance rate observed for *A. salmonicida* (multiple antibiotic resistance index of 0.25). Of the 21 isolates, 11 were biofilm producers, and 10 of them were strong biofilm producers (SBPs). The SBPs presented increased tolerance to chlorine disinfection when compared with their planktonic counterparts. In order to elucidate the mechanisms underlying biofilm tolerance to chlorine and support the importance of preventing biofilm assembly in water reservoirs, further research is required.

## 1. Introduction

*Aeromonas* spp. are Gram-negative rod-shaped bacteria widely distributed in different aquatic environments, such as saline water, freshwater, wastewater and even drinking water [1,2]. *Aeromonas* spp. are regarded as fish pathogens, but their ability to infect a wider range of hosts, including reptiles, crustaceans, amphibians and humans, is recognized [3]. *A. hydrophyla*, *A. caviae* and *A. veronii* are the species most likely to infect humans. In human beings, clinical manifestations can range from gastrointestinal tract disorder (e.g., travelers’ diarrhea) to heart, skin, eye and other organ infections, and even lethal sepsis [4].

Multidrug resistance has been reported for *Aeromonas* spp. isolated from fish and different water sources (e.g., wastewater treatment effluents, rivers, etc.) [5,6,7]. It has been suggested that these microorganisms can be used as ecological indicators of water pollution since they harbor antibiotic-resistance genes obtained, for example, from wastewater effluents [5]. Antibiotic resistance among *Aeromonas* spp. represents a major problem for aquaculture because of their ability to infect economically relevant species of prawns and fish [6]. Intrinsic resistance, mutations in specific genes, efflux pumps and horizontal gene transference are considered relevant mechanisms for the emergence of antibiotic resistance among *Aeromonas* spp. and were recently reviewed by Carusi and colleagues [8]. The increased use of antibiotics in human, animal and plant health and the ability of microorganisms to assemble biofilms [9] contribute to a major public health concern regarding antibiotic treatment failure.

In the environment, the majority of microorganisms do not persist as discrete planktonic forms but are instead associated with a matrix within a structure known as a biofilm [10]. Biofilm-embedded microorganisms are more resilient to pH and temperature shifts, nutrient deprivation and other stress factors [10,11]. The presence of biofilms in water supply systems can decrease the efficiency of disinfection procedures, such as chlorination [12]. The widespread use of chlorination has promoted the selection of chlorine-resistant bacteria, which could represent an issue for public health [13]. Although the mechanisms involved in chloride resistance are not fully elucidated, it is known that the secretion of extracellular matrix (EPS) plays a role by limiting the exposure of bacteria to oxidative agents [14,15].

The major goals of the present work are to compare the antibiotic susceptibility profiles of *Aeromonas* spp. isolated from different freshwater bodies, their ability to assemble biofilms and the role played by biofilms in *Aeromonas* resilience to water chlorination.

## 2. Results

### 2.1. Characterization of Aeromonas Isolates

In the present study, 21 environmental isolates of *Aeromonas* spp. from freshwater and biofilm samples were used (Table 1): 13/21 isolates from natural freshwater; 4/21 isolates from anthropogenically treated freshwater; and 2/21 isolates each from biofilms present on natural or anthropogenic freshwater reservoirs(Table 1). All natural freshwater samples were collected from dams located in *Alentejo*. Samples from *Monsaraz* (Mz), *Mourão* (Mo) and *Amieira* (Am) were collected during 2021 once per season [16] or during the summer of 2016 for samples from *Monte da Rocha* (MR) and *Alvito* (Al) [17]. All the samples from anthropogenic freshwater reservoirs were collected during 2016 in an urban park located in Lisbon.

The isolates were first identified using Vitek-MS and then by *16S rRNA* sequencing. The same identification at the species level was achieved for 5/21 isolates (24%) using both methods; one of the species rendered by Vitek-MS for 3/21 (14%); and different species for 13/21 isolates (62%). Since the *16S rRNA* sequencing has a higher discriminatory ability, we decided to adopt the classification rendered by this method (Table 1). Five species of *Aeromonas* were identified: *A. hydrophila* (1/21) and *A. hydrophila/veronii* (1/21); *A. popoffii* (1/21); *A. media* (1/21); *A. salmonicida* (3/21); and *A. veronii* (14/21). *A. veronii* was the predominant species in all samples and was the only species isolated from water and biofilms in the treated water reservoir located at Lisbon’s urban park. In natural water reservoirs, higher species diversity was observed for water samples, although from biofilms, only one species, *A. veronii*, was identified.

Next, the antimicrobial susceptibility profile of *Aeromonas* spp. was determined (Table 2). *A. popoffii* (Am-W/21/06), *A. media* (Mo-W/21/10) and *A. hydrophila* (Mz-W/21/60) were pan-susceptible. *A. hydrophila/veronii* (Mo-W/21/65) and one isolate of *A. salmonicida* (Mz-W/21/18) were susceptible to all tested antibiotics except imipenem (IMP10); for each, the isolate was classified as “Susceptible, increased exposure”, which was previously classified as intermediate. In order to simplify, in this work, we adopted the intermediate classification for the “Susceptible, increased exposure” category, which can be interpreted as values between the susceptible and the resistant breakpoints. The other two *A. salmonicida* isolates were resistant to two (Mo-W/21/15 FOX and IMP) or three antibiotics (Mo-W/21/09 CAZ, FOX and IMP), and they were the most resistant isolates of the study. Among *A. veronii* isolates, four profiles could be found: pan-susceptible (1/14 isolates), with intermediate susceptibility to one, antibiotic—CAZ10 or IMP10 (3/14 isolates); resistant to one antibiotic—IMP10 or CN30 (6/14 isolates); and resistant to two antibiotics (4/14 isolates). For the last case, two combinations were observed, IMP10 and MEM30 (3/4 isolates) and FOX30 and IMP10 (1/4 isolates). The antibiotic susceptibility profiles of *A. veronii* isolated from water and biofilm were similar.

When determining the prevalence of resistance within the same species, the multiple antibiotic resistance index (MAR_index_) was higher for *A. salmonicida* (three isolates) with a value of 0.250 than for *A. veronii* (14 isolates), for which a value of 0.125 was calculated (Table 3). For both *Aeromonas* spp., IMP registered the highest resistance rate. Then, despite the relatively small sample size, which is difficult to extrapolate from, *A. salmonicida* isolates presented a higher resistance rate to cephalosporins and were susceptible to aminoglycoside gentamicin (CN), whereas the opposite profile was observed for *A. veronii*.

### 2.2. Biofilm Assembly

The ability of the 21 *Aeromonas* spp. isolates to assemble biofilms in vitro after 24 h of incubation at 37 °C was evaluated. All isolates of *A. salmonicida* (n = 3), *A. hydrophila* (n = 1), *A. hydrophila/veronii* (n = 1), *A. media* (n = 1) and *A. popoffii* (n = 1) were considered non-biofilm producers (NBP) according to Stepanovics’ classification [18]. Among *A. veronii* isolates (n = 14), three (21.4%) were classified as NBP; one (7.1%) as a weak biofilm producer (WBP); and the remaining ten (71.4%) were classified as strong biofilm producers (SBPs), as shown in Table 4. The ability to assemble biofilms and lyse red blood cells could be considered a virulence factor. In addition, there are reports of a link between biofilm formation and the ability of bacteria to move on solid surfaces [19]. For this reason, the ability of the different isolates to move by swimming and swarming and to lyse red blood cells was evaluated. The obtained results are shown in Table 4. The majority of the isolates, with the exception of one isolate of *A. salmonicida* (Mo-W/21/15) and *A. hydrophila/veronii* (Mo-W/21/15), were able to move by swimming. An almost opposite result was observed for swarming movement, with only one *A. salmonicida* (Mo-W/21/09) and three *A. veronii* isolates (MR-W/16/34, UP-B/16/50 and U-B/16/53) being able to show a positive result. The obtained results did not show a good correlation between the ability to move and the ability to assemble biofilms, neither in vitro nor in natural environments.

In addition to the ability to assemble biofilm, the ability to lyse red blood cells could be regarded as a virulence factor. All isolates of *A. salmonicida* (n = 3), *A. hydrophila* (n = 1), *A. hydrophila*/*veronii* (n = 1) and *A. popoffii* (n = 1) were hemolytic. Among *A. veronii* isolates, only 57.1% of the isolates (n = 8) were hemolytic. The remaining six isolates of *A. veronii* and the *A. media* isolate did not show hemolytic activity (Table 4).

### 2.3. Chlorination

**Figure 1:**
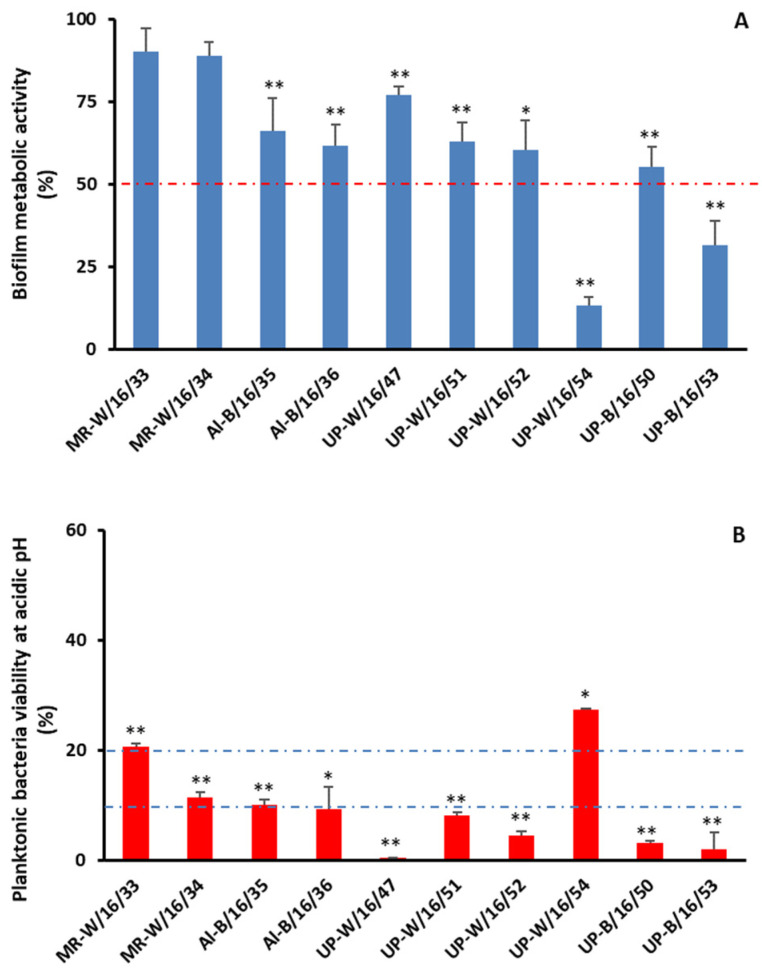
*Aeromonas* spp. persistence. Metabolic activity was assessed by a MTT assay of *Aeromonas veronii* biofilms, which was used to evaluate its susceptibility to chlorination (**A**), whereas the enumeration of colony-forming units (CFUs) was used to evaluate the susceptibility of planktonic counterparts to acidic pH (**B**). *p* < 0.05 (*) was considered statistically significant; *p* < 0.01 (**) was considered highly significant.

Biofilms might function as reservoirs of potential infectious agents that could cause disease in humans and animals. Chlorine, either in residual concentrations or in higher concentrations, could be used to preserve tap water microbiological quality or as a disinfection agent, respectively. Here, we decided to evaluate the ability of chlorine in higher concentrations to control *Aeromonas* spp. organized within biofilms. The 10 isolates of *A. veronii* considered SBPs were selected for this assay. After 3 h of treatment with 10 mg/L free chlorine, a decrease of more than 50% in bacterial metabolic activity compared with the control was found for only 2/10 isolates (Figure 1A). In addition, no statistical difference was found for the metabolic activity of biofilms assembled by MR-W/16/33 and MR-W/16/34 in the presence of high chlorine compared to the control, supporting the ability of biofilms to protect against this disinfection procedure. It was not possible to establish a link between either previous exposure to chlorine and resistance to it (*A. veronii* isolated from natural waters exhibited higher tolerance levels to chlorine than *A. veronii* isolated from treated water) or biofilm assembly in the environment (*A. veronii* isolated from water were more tolerant to chlorine than *A. veronii* isolated from biofilms). These results led us to think that planktonic forms of *A. veronii* isolates might differ in their susceptibility to chlorine. Planktonic bacteria’s susceptibility to residual-free chlorine concentrations (0.2 mg/L) in the range allowed by Portuguese law to be present in tap water [20] and to high free chlorine concentrations (10 mg/L) used in disinfection procedures was determined. In both conditions, after 3 h of exposure, bacteria were eradicated. This result could be surprising since it was possible to isolate *Aeromonas* spp. in treated water samples with residual levels of chlorine (≤0.16 mg/L—Table 5). Since, in our experimental setup, an acidic pH was used, and the pH of natural and anthropogenic waters ranged between seven and eight, we evaluated the effect of acidic pH on bacteria (Figure 1B). Indeed, a statistically significant decrease in colony-forming unit (CFU) counts was found for all isolates in acidic conditions compared with the control. For 2/10 isolates, after 3 h, only 20% of the bacteria were viable, and for 8/10 isolates, 10% or less were viable (Figure 1B).

## 3. Discussion

*Aeromonas* spp. are ubiquitous environmental microorganisms but also etiological agents of human diseases. An accurate identification of etiological agents is crucial for correct diagnosis and successful treatment. For this reason, we started by identifying the environmental isolates of *Aeromonas* using the methods available at our clinical microbiology laboratory: the Vitek-MS system and *16S rRNA* sequencing. A certain discrepancy was noticed between the results obtained with the two methods, with multiple possibilities of classification for five isolates using the Vitek-MS system and only one isolate with *16S rRNA* sequencing. Of note, the nine cases of discrepant identifications between the two methods happened for the closely related species of *A. sobria* and *A. veronii*. These results show that the accurate identification of *Aeromonas* at the species level could be a challenge. Despite the higher cost, the need for dedicated trained staff and proper implementation, the adoption of other methodologies described as more accurate for species identification—such as housekeeping gene (e.g., *gyrB*, *rpoB*) sequencing or even whole-genome sequencing for critical cases—should be considered for diagnosis purposes [21,22,23]. Here, we decided to adopt an identification method based on *16S rRNA* sequencing that identified five different species of *Aeromonas*, *A. salmonicida*, *A. hydrophila*, *A. media*, *A. popoffii* and *A. veronii*, from freshwater samples. From biofilms present in natural and anthropogenic freshwater bodies, only the mesophilic species *A. veronii* was identified. In our opinion, this observation might derive from the sample size (only four biofilm samples) and not from a special propensity of *A. veronii* to assemble biofilms. Despite the limitations of the sample size in the analyzed water bodies, *A. veronii* was the most prevalent species of *Aeromonas*, as in other studies [24,25]. Water temperature has been described to significantly affect the abundance of *Aeromonas* in water bodies [25]. This was not a quantitative study, but different *Aeromonas* species were isolated in a considerably wide range of water temperatures (14–33 °C, Table 5), suggesting that temperature might affect the abundance more than the diversity of *Aeromonas* spp.

In order to evaluate a potential health risk, antibiotic susceptibility and other potential virulence factors (e.g., biofilm assembly, hemolysis) were evaluated at 37 °C (human body temperature). Multidrug-resistant (MDR) bacteria, defined as those resistant to three or more antibiotics, were not abundant in our sample. Only one isolate of *A. salmonicida* (Mo-W/21/09) was MDR, showing resistance to three antibiotics (Table 2). *Aeromonas salmonicida* was also the only species exhibiting a MAR_index_ ≥ 0.2 (Table 3), suggesting that the isolates originated from a high-risk source of contamination [7]. Despite this fact, 23.8% (5/21) of the isolates were resistant to two antibiotics, including a carbapenem (imipenem—IMP) that is still a reserve antibiotic [26]. This fact could be challenging in cases of infections that are refractory to antibiotic treatment since another 4/21 and 6/21 isolates displayed intermediate resistance to IMP and meropenem (MEM), respectively. A more detailed study aimed at elucidating the molecular mechanisms responsible for the emergence of resistance to these antibiotics should be conducted in the future. Of note, with the exception of the MDR isolate that displayed intermediate resistance to the fluoroquinolone ciprofloxacin (CIP), all isolates were susceptible to this antibiotic, in good agreement with previous findings [4].

Next, the hemolytic activity of the *Aeromonas* spp. isolates was evaluated. All *Aeromonas* spp., except *A. media*, have isolates with hemolytic activity (Table 4). *Aeromonas* spp.’s hemolytic activity is a virulence factor mediated by hemolytic toxins such as aerolysin and hemolysin [27]. For this reason, a future study would be interesting to evaluate the expression of the genes responsible for aerolysin and hemolysin at different conditions in order to elucidate their role in the virulence of these isolates.

Lastly, the ability of *Aeromonas* spp. to assemble biofilms was evaluated. More than half of the isolates (11/21) exhibited this ability, with one being a WBP and the remaining ten being SBPs. No straightforward relationship between biofilm assembly, hemolytic activity or antibiotic resistance was identified. For this reason, we did not perform an antibiotic susceptibility test for the biofilms. Instead, the ability of *Aeromonas* to move on solid media by swimming and swarming, previously described to be related to biofilm assembly, was evaluated [19,28]. For our *Aeromonas* isolates, it was not possible to establish this connection.

Biofilms are known to protect microorganisms from external aggressions (e.g., antimicrobials, UV, etc.) and are the most common form of microorganism presentation, although for study proposes, planktonic forms are most often used [29,30]. Therefore, we decided to evaluate the efficacy of biofilms in protecting microorganisms from water disinfection with chlorine. Indeed, only for two of the ten SBP isolates was a decrease in bacteria metabolic activity higher than 50% observed after 3 h treatment with chlorine in comparison with the control (untreated biofilms) (Figure 1A). This result argues in favor of the protective role of biofilms since planktonic forms of the same bacteria were eradicated when submitted to the same conditions or even to the residual concentrations of chlorine allowed in tap water. This result could be partially explained by exposure to low pH (Figure 1B), although acid tolerance has been described for certain species of *Aeromonas* [31,32]. The tolerance of *Aeromonas* spp. to chlorine has been previously described [33,34], and the results presented here for biofilm tolerance show the need to prevent biofilm assembly in water supply systems in order to ensure water safety. The elucidation of the mechanisms underlying chlorine tolerance might be detrimental to the development of more effective disinfection procedures.

## 4. Materials and Methods

### 4.1. Sampling Collection

Water samples were collected as previously described with slight modifications [35]. Briefly, one liter of superficial water was collected using a sterile glass bottle from an ornamental fountain located at an urban park (UP) in *Lisboa* (anthropogenic source) or at 3 dams in *Alentejo*, namely, *Alqueva*, *Monte da Rocha* and *Alvito*. Alqueva samples were collected at three locations: *Monsaraz* (Mz—N 38.43455° W 7.35037°), *Mourao* (Mo—N 38.36775° W 7.35582°) and *Amieira* (Am—N 38.27716° W 7.53315°). For *Monte da Rocha* (MR—N 37.72763°, W −8.29150°) and *Alvito* (Al—N 38.28226°, W −7.91598°), the collection was conducted at only one location. At the same locations, a swab was used to collect biofilm samples from a 10 cm^2^ area, which were introduced to 10 mL of phosphate buffer saline (PBS, Lonza, Basel, Switzerland). Samples were transported in refrigerated containers protected from light and processed upon arrival at the laboratory. Water temperature and pH were assessed in the field using a probe and a multiparameter apparatus from Lovibond^®^ (Tintometer GmbH, Dortmund, Germany), respectively, according to the manufacturer’s instructions.

### 4.2. Microorganism Isolation and Identification

Water samples were homogenized by inverting the recipient several times before 10 mL of the sample was filtrated through membrane filters with 0.45 μm pore diameters (Merck-Millipore, Darmstadt, Germany) using a filtration slant (Merck-Millipore). The membranes were then transferred either to non-selective (Mueller–Hinton—MH) agar (from Oxoid, Basingstoke, UK) or selective (MacConkey from Oxoid) solid culture media and incubated at 30 °C and 37 °C for 24 h. Bacterial identification was first performed using Vitek-MS systems (bioMerieux, Marcy l’Etoile, France). Briefly, a homogeneous microbial suspension was prepared from overnight cultures in 0.45% sodium chloride solution adjusted to a turbidity of 0.5 McFarland (~1.5 × 10^8^ colony-forming units (CFUs)/mL). The microbial suspension was further processed according to the manufacturer’s instructions.

One inoculation loop of fresh bacterial culture was removed from the culture plate, and DNA was extracted using the Qiagen DNeasy Blood & Tissue Kit (Qiagen, Hilden, Germany) according to the supplier’s instructions. Identification of *Aeromonas* isolates to the species level was confirmed by *16S rRNA* sequencing. A 976 bp section of the *16S rRNA* gene was RT-PCR-amplified using the following primers: F-AGAGTTTGATCMTGGCTCAG and R-GTAAGGTTCTKCGCGTTGC. An in-house RT-PCR using SyberGreen (Roche Diagnostics, Manheim, Germany) was performed in the following conditions: denaturation (10 min at 95 °C), hybridization (5 s at 63 °C, 40 cycles) and elongation (39 s at 72 °C), followed by a melting curve (30 s at 40 °C) using a CFX OPUS apparatus (Bio-Rad, Hercules, CA, USA), and fluorescence was acquired at 530 nm. The amplification products were purified using Exosap (Applied Biosystems, Foster City, CA, USA) according to the manufacturer’s instructions. The PCR for sequencing was performed with the primers described above in the following conditions: denaturation (30 s at 96 °C), followed by 25 cycles of amplification (10 s at 96 °C; 10 s at 50 °C; 4 min at 60 °C). The samples were submitted to Sanger Sequencing, and the obtained sequences were analyzed using the BioEdit Sequence Alignment Editor 7.2.5. software (Bioedit Company, Manchester, UK) and compared with known sequences in GenBank (http://www.ncbi.nlm.nih.gov (accessed on 2 November 2020) using the BLASTN 2.14.1+ algorithm (http://blast.ncbi.nlm.nih.gov/Blast.cgi (accessed on 2 November 2020)).

### 4.3. Antimicrobial Susceptibility Testing

The antimicrobial activity was tested using the disk diffusion method described by the EUCAST Guidelines. Briefly, a bacterial suspension adjusted to a turbidity of 0.5 McFarland was inoculated on MH-agar; challenged with the following antibiotics (Oxoid): ceftazidime (CAZ 10 μg), ciprofloxacin (CIP 5 μg), levofloxacin (LEV 5 μg), trimethoprim-sulfamethoxazole (STX 25 μg), cefoxitin (FOX 30 μg), imipenem (IMP 10 μg), meropenem (MEM 10 μg) and gentamicin (CN 30 μg); and incubated at 35 ± 1 °C overnight before inhibition halos were read. The results were interpreted according to EUCAST guidelines for *Aeromonas* spp. (CAZ, CIP, LEV and STX) when available [36] and according to Skwor and colleagues for *Enterobacteriaceae* (FOX, IMP, MEM and CN) [7].

### 4.4. Biofilm Assay

The assay was performed in triplicate using 96-well flat-bottomed cell culture plates (Nunc, New York, NY, USA), as described previously [37] with slight modifications. Briefly, bacterial suspensions at a final concentration of 10^8^ CFU/mL were prepared in PBS from overnight cultures in MH-agar and ten-fold diluted in MH broth (Oxoid). In total, 200 μL was distributed to each well with MH broth used as the negative control. The plates were incubated at 37 °C to allow for biofilm formation for 24 h. The well content was removed, and each well was vigorously washed three times with sterile distilled water. The attached bacteria were stained for 15 min with 100 µL of 1.4% crystal violet at room temperature; washed with distilled water three times; and allowed to dry at room temperature. The crystal violet was dissolved in 95% ethanol (Merck, Darmstadt, Germany), and the optical density at 570 nm was read using a SpectraMax 340 PC (Molecular Devices, Sunnyvale, CA, USA). The results of optical density readings were interpreted according to Stepanovic and coauthors [18]. Briefly, the cut-off value (ODc) was defined as three standard deviations (SDs) above the mean OD of the blank. Based on the previously calculated OD values (ODs) for different conditions, the results were interpreted as follows: ODs ≤ ODc, non-biofilm producer (NBP); ODc < Ods ≤ 2ODc, weak biofilm producer (WBP); 2 ODc < ODs ≤ 4 ODc, moderate biofilm producer (MBP); 4 ODc < ODs, strong biofilm producer (SBP). At least three independent experiments were performed.

### 4.5. Mobility Assays and Hemolytic Activity

Bacterial isolates were grown overnight on MH-agar. A sterile 1.5 μL loop was used to transfer and inoculate them into the center of motility agar to evaluate swimming (1% tryptone (Oxoid), 0.5% NaCl (Merck), 0.25% agar (Oxoid)) or swarming (1% tryptone, 0.5% NaCl, 0.6% agar) in freshly grown bacterial colonies. The plates were incubated face-up for 18–24 h at 30 °C, and motility was assessed by examining the migration of bacteria through the agar from the center toward the periphery of the plate [38].

In order to evaluate bacterial hemolytic activity, bacterial isolates were transferred to blood agar (bioMerieux) and incubated for 18–24 h at 37 °C. The presence of a transparent halo around bacterial growth was considered positive for hemolytic activity.

### 4.6. Chlorination

#### 4.6.1. Biofilms

The *Aeromonas* isolates classified as SBPs were selected for a chlorination assay. Bacteria were allowed to assemble biofilm for 24 h as described in Section 4.4. The well content was removed, and each well was vigorously washed three times with sterile distilled water. Biofilms were then incubated at room temperature (25 °C) at 200 rotations per minute (rpm), protected from light for 3 h either in 200 μL of spring water (control—*Águas doCaramulo*, *Varzielas*, *Portugal*) or in 200 μL of chlorination mixture. This mixture was prepared by adding 14% NaOCl (VWR, Radnor, PA, USA) to spring water followed by acidification to pH 3 and the titration of free chlorine using a photometric method and the Lovibond^®^ Water Testing kit, according to the manufacturer’s instructions (Tintometer GmbH, Dortmund, Germany). The volume of NaOCl solution was adjusted in order to obtain a free chlorine concentration of approximately 10 mg/L. At the end of the assay, 24% ammonia solution (Merck) was added to neutralize the mixture.

Biofilm viability was determined by incubating 10 μL of MTT (Sigma-Aldrich, Sintra, Portugal) solution (5 mg/mL) per 100 μL of spring water for 4 h at RT with shaking (200 rpm). Afterward, the MTT solution was removed, and 100 μL of DMSO (Sigma) was added to each well to dissolve the formazan crystals for 30 min at room temperature in the dark. Absorbance was measured at 570 nm using a spectrophotometer (SpectraMax 340 PC), as previously described [39]. Cell viability (percentage), assessed by mitochondrial activity (MTT), was calculated as the ratio between the mean absorbance of the treated (chlorination) and control samples. The results are expressed as the mean value of at least 3 independent experiments. Statistical significance was assessed by Student’s two-tailed *t*-test. *p* < 0.05 (*) was considered statistically significant, and a *p* < 0.01 (**) was considered highly significant.

#### 4.6.2. Planktonic Bacteria

For the same *Aeromonas* isolates used in Section 4.6.1, suspensions at a final concentration of 10^8^ CFU/mL were prepared in PBS from overnight cultures in MH-agar and ten-fold diluted in MH broth (Oxoid). One milliliter of bacterial suspension was transferred to a sterile Eppendorf and centrifuged for 10 min at 2500 rpm (Jouan, Thermo Fisher Scientific, Waltham, MA, USA). the supernatant was discarded, and the pellet was suspended in spring water (control: Ctr), a low chlorination mixture (0.2 mg/L residual-free chlorine—low chlorine), a high chlorination mixture (10 mg/L free chlorine—high chlorine) and acidified spring water (pH control). The Eppendorfs were incubated at room temperature in the dark, 200 rpm for 3 h. All conditions except the Ctr were neutralized as described in Section 4.6.1. The Eppendorfs were centrifuged (2500 rpm, 10 min); the supernatant was discarded; and the pellet was resuspended in 1 mL of spring water. The suspension was serially diluted in water and plated in MH-agar prior to overnight incubation at 37 °C. The CFUs were enumerated, and the bacterial survival was calculated as a percentage of the control (bacteria incubated with water). The results are expressed as the mean value of at least three independent experiments performed in triplicate. Statistical significance was assessed by Student’s two-tailed *t*-test. *p* < 0.05 (*) was considered statistically significant, and *p* < 0.01 (**) was considered highly significant.

## Figures and Tables

**Table 1:** Identification of *Aeromonas* spp. by different methods.

Source	ID	Vitek-MS	*16S rRNA*
Water	Mz-W/21/18	*A. salmonicida/bestiarium*	*A. salmonicida*
(Natural)	Mz-W/21/60	*A. sobria*	*A. hydrophila*
	Mz-W/21/58	*A. sobria*	*A. veronii*
	Mo-W/21/09	*A. salmonicida/bestiarium*	*A. salmonicida*
	Mo-W/21/15	*A. salmonicida/bestiarium*	*A. salmonicida*
	Mo-W/21/65	*A. sobria*	*A. hydrophila/veronii*
	Mo-W/21/10	*A. media*	*A. media*
	Am-W/21/06	*A. salmonicida/bestiarium*	*A. popoffii*
	Am-W/21/53	*A. sobria*	*A. veronii*
	Am-W/21/05	*A. veronii*	*A. veronii*
	Am-W/21/07	*A. veronii*	*A. veronii*
	MR-W/16/33	*A. veronii*	*A. veronii*
	MR-W/16/34	*A. sobria*	*A. veronii*
Biofilm	Al-B/16/35	*A. sobria*	*A. veronii*
(Natural)	Al-B/16/36	*A. sobria*	*A. veronii*
Water	UP-W/16/47	*A. sobria*	*A. veronii*
(Anthropogenic)	UP-W/16/51	*A. hydrophila/caviae*	*A. veronii*
	UP-W/16/52	*A. veronii*	*A. veronii*
	UP-W/16/54	*A. sobria*	*A. veronii*
Biofilm	UP-B/16/50	*A. sobria*	*A. veronii*
(Anthropogenic)	UP-B/16/53	*A. sobria*	*A. veronii*

**Table 2:** Antibiotic susceptibility profiles of *Aeromonas* spp.

Source	ID	CAZ10	CIP5	LEV5	STX25	FOX30	IMP10	MEM10	CN30
Water	Mz-W/21/18	S	S	S	S	S	I	S	S
(Natural)	Mz-W/21/60	S	S	S	S	S	S	S	S
	Mz-W/21/58	S	S	S	S	S	R	S	S
	Mo-W/21/09	R	I	S	S	R	R	S	S
	Mo-W/21/15	S	S	S	S	R	R	S	S
	Mo-W/21/65	S	S	S	S	S	I	S	S
	Mo-W/21/10	S	S	S	S	S	S	S	S
	Am-W/21/06	S	S	S	S	S	S	S	S
	Am-W/21/53	S	S	S	S	S	R	I	S
	Am-W/21/05	I	S	S	S	S	S	S	S
	Am-W/21/07	I	S	S	S	S	S	S	S
	MR-W/16/33	S	S	S	S	S	S	S	S
	MR-W/16/34	S	S	S	S	R	R	S	S
Biofilm	Al-B/16/35	S	S	S	S	S	I	S	S
(Natural)	Al-B/16/36	S	S	S	S	S	R	S	S
Water	UP-W/16/47	S	S	S	S	S	S	S	R
(Anthropogenic)	UP-W/16/51	S	S	S	S	S	R	I	R
	UP-W/16/52	S	S	S	S	S	I	I	R
	UP-W/16/54	S	S	S	S	S	R	I	R
Biofilm	UP-B/16/50	S	S	S	S	S	S	I	R
(Anthropogenic)	UP-B/16/53	S	S	S	S	S	R	I	R

CAZ10: ceftazidime, 10 µg; CIP5: ciprofloxacin, 5 µg; LEV5: levofloxacin, 5 µg; STX25: trimethoprim–sulfamethoxazole, 25 µg; FOX30: cefoxitin, 30 µg; IMP10: imipenem, 10 µg; MEM10: meropenem, 10 µg; CN30: gentamicin, 30 µg; S: susceptible; I: intermediate—the obtained value was between the S and R breakpoints; R: resistant; ID: isolate identification.

**Table 3:** Resistance profile of *Aeromonas* populations.

Bacteria (No. of Isolates)	% (No.) of Isolates Resistant to an Antibiotic ^a^				MAR_index_ ^b^
	CAZ10	FOX30	IMP10	CN30	
*A. salmonicida*	33% (1)	67% (2)	67% (2)	0% (0)	0.250
(n = 3)					
*A. veronii*	0% (0)	7.2% (1)	50% (7)	43% (6)	0.125
(n = 14)					

^a^ Only antibiotics for which resistance was observed are included in the table. CAZ10: ceftazidime, 10 µg, FOX30: cefoxitin, 30 µg; IMP10: imipenem, 10µg; CN30: gentamicin, 30 µg. ^b^ The median multiple antibiotic resistance (MAR) index among the isolates of one species.

**Table 4:** Biofilm assembly and hemolytic activity of *Aeromonas* spp.

Species (No. of Isolates)	ID	Biofilm ^a^	Swimming ^b^	Swarming ^c^	Hemolysis ^d^
*A. salmonicida* (3)	Mz-W/21/18	NBP	+	-	+
	Mo-W/21/09	NBP	+	+	+
	Mo-W/21/15	NBP	-	-	+
*A. hydrophila* (1)	Mz-W/21/60	NBP	+	-	+
*A. hydrophila/veronii* (1)	Mo-W/21/65	NBP	-	-	+
*A. media* (1)	Mo-W/21/10	NBP	+	-	-
*A. popoffii* (1)	Am-W/21/06	NBP	+	-	+
*A. veronii*	Mz-W/21/58	WBP	+	-	+
(14)	Am-W/21/53	NBP	++	-	+
	Am-W/21/05	NBP	+	-	-
	Am-W/21/07	NBP	+	-	-
	MR-W/16/33	SBP	++	-	+
	MR-W/16/34	SBP	++	+	-
	Al-B/16/35	SBP	+	-	+
	Al-B/16/36	SBP	+	-	+
	UP-W/16/47	SBP	+	-	-
	UP-W/16/51	SBP	++	-	+
	UP-W/16/52	SBP	+	-	+
	UP-W/16/54	SBP	++	-	+
	UP-B/16/50	SBP	+	+	-
	UP-B/16/53	SBP	+	+	-

^a^ NBP: non-biofilm producer; WBP: weak biofilm producer; SBP: strong biofilm producer. ^b^ Swimming is determined as migration from the source of inoculation in LB broth containing 0.25% agar after 16 to 24 h at 30 °C. ++, spreading zone ≥ 7 cm from the central point of inoculation; +, spreading zone ≥ 2 cm but <7 cm from the inoculation point; -, growth at the inoculum site but no movement. ^c^ Swarming is determined as surface migration on Eiken agar swarm plates (0.6% agar) after 16 to 24 h at 30 °C. +, surface motility zone ≥ 3 cm but <8 cm; -, growth at the inoculum site but no surface movement [19]. ^d^ + present, - absent. ID: isolate identification.

**Table 5:** Water properties.

Bacteria			Water	
Species	ID	pH	Chlorine (mg/L)	Temperature (°C)
*A. veronii*	MR-W/16/33	6.60	---	27.0
	MR-W/16/34	6.60	---	27.0
	Al-B/16/35	7.10	---	33.0
	Al-B/16/36	7.10	---	33.0
	UP-W/16/47	7.93	---	14.7
	UP-W/16/51	8.14	0.16	17.0
	UP-W/16/52	8.14	0.10	17.0
	UP-W/16/54	7.91	0.10	14.0
	UP-B/16/50	8.01	---	17.0
	UP-B/16/53	7.91	---	14.0

--- Chlorine < 0.05 mg/L; ID: isolate identification.

## Data Availability

Data are contained within the article.

## References

[1] Fernández-Bravo A., Figueras M.J. (2020). An Update on the Genus *Aeromonas*: Taxonomy, Epidemiology, and Pathogenicity. Microorganisms.

[2] van der Wielen P.W.J.J., Bakker G., Atsma A., Lut M., Roeselersd G., de Graaf B. (2016). survey of indicator parameters to monitor regrowth in unchlorinated drinking water. Environ. Sci. Water Res. Technol..

[3] Janda J.M., Abbott S.L. (2010). The genus *Aeromonas*: Taxonomy, pathogenicity, and infection. Clin. Microbiol. Rev..

[4] Pessoa R.B.G., de Oliveira W.F., Correia M.T.S., Fontes A., Coelho L.C.B.B. (2022). *Aeromonas* and Human Health Disorders: Clinical Approaches. Front. Microbiol..

[5] Conte D., Palmeiro J.K., Bavaroski A.A., Rodrigues L.S., Cardozo D., Tomaz A.P., Camargo J.O., Dalla-Costa L.M. (2021). Antimicrobial resistance in *Aeromonas* species isolated from aquatic environments in Brazil. J. Appl. Microbiol..

[6] Nhinh D.T., Le D.V., Van K.V., Huong Giang N.T., Dang L.T., Hoai T.D. (2021). Prevalence, Virulence Gene Distribution and Alarming the Multidrug Resistance of *Aeromonas hydrophila* Associated with Disease Outbreaks in Freshwater Aquaculture. Antibiotics.

[7] Skwor T., Stringer S., Haggerty J., Johnson J., Duhr S., Johnson M., Seckinger M., Stemme M. (2020). Prevalence of potentially pathogenic antibiotic-resistant *Aeromonas* spp. in treated urban wastewater effluents versus recipient riverine populations: A 3-year comparative study. Appl. Environ. Microbiol..

[8] Carusi J., Kabuki D.Y., de Seixas Pereira P.M., Cabral L. (2024). *Aeromonas* spp. in drinking water and food: Occurrence, virulence potential and antimicrobial resistance. Food Res. Int..

[9] Martinez J.L. (2009). Environmental pollution by antibiotics and by antibiotic resistance determinants. Environ. Pollut..

[10] Ansari M.I., Schiwon K., Malik A., Grohmann E., Malik A., Grohmann E. (2012). Biofilm formation by environmental bacteria. Environmental Protection Strategies for Sustainable Development.

[11] Hall-Stoodley L., Costerton J.W., Stoodley P. (2004). Bacterial biofilms: From the natural environment to infectious diseases. Nat. Rev. Microbiol..

[12] Koizumi Y., Ichijo T., Uchii K., Nasu M. (2023). Changes in bacterial diversity and community structure in drinking water distribution system revealed by high throughput sequencing. J. Microorg. Control.

[13] Lu Y.W., Liang X.X., Wang C.Y., Chen D., Liu H. (2023). Synergistic nanowire-assisted electroporation and chlorination for inactivation of chlorine-resistant bacteria in drinking water systems via inducing cell pores for chlorine permeation. Water Res..

[14] Luo L.W., Wu Y.H., Chen G.Q., Wang H.B., Wang Y.H., Tong X., Bai Y., Xu Y.Q., Zhang Z.W., Ikuno N. (2022). Chlorine-resistant bacteria (CRB) in the reverse osmosis system for wastewater reclamation: Isolation, identification and membrane fouling mechanisms. Water Res..

[15] Cho M., Kim J., Kim J.Y., Yoon J., Kim J.H. (2010). Mechanisms of *Escherichia coli* inactivation by several disinfectants. Water Res..

[16] Raposo A., Mansilha C., Veber A., Melo A., Rodrigues J., Matias R., Rebelo H., Grossinho J., Cano M., Almeida C. (2022). Occurrence of polycyclic aromatic hydrocarbons, microplastics and biofilms in Alqueva surface water at touristic spots. Sci. Total Environ..

[17] Sousa M., Morgado P., Rodrigues J., Matias R., Nogueira I., Jordao L. (2019). Caracterização da população bacteriana em barragens na bacia hidrografica do Sado. Bol. Epidemiol. Obs..

[18] Stepanovic S., Vukovic D., Hola V., di Bonaventura G., Djukic S., Cirkovic I., Ruzicka F. (2007). Quantification of Biofilm in Microtiter Plates: Overview of Testing Conditions and Practical Recommendations for Assessment of Biofilm Production by Staphylococci. APMIS.

[19] Kirov S.M., Tassell B.C., Semmler A.B., O’Donovan L.A., Rabaan A.A., Shaw J.G. (2002). Lateral flagella and swarming motility in *Aeromonas* species. J. Bacteriol..

[20] Decreto-Lei n° 152/2017, de 7 de Dezembro. https://diariodarepublica.pt/dr/detalhe/decreto-lei/152-2017-114315242.

[21] Bertran X., Rubio M., Gómez L., Llovet T., Muñoz C., Navarro F., Miro E. (2021). Taxonomic Identification of Different Species of the Genus *Aeromonas* by Whole-Genome Sequencing and Use of Their Species-Specific β-Lactamases as Phylogenetic Markers. Antibiotics.

[22] Shin H.B., Yoon J., Lee Y., Kim M.S., Lee K. (2015). Comparison of MALDI-TOF MS, housekeeping gene sequencing, and *16S rRNA* gene sequencing for identification of *Aeromonas* clinical isolates. Yonsei Med. J..

[23] Soler L., Yáñez M.A., Chacon M.R., Aguilera-Arreola M.G., Catalán V., Figueras M.J., Martínez-Murcia A.J. (2004). Phylogenetic analysis of the genus *Aeromonas* based on two housekeeping genes. Int. J. Syst. Evol. Microbiol..

[24] Dhanapala P.M., Kalupahana R.S., Kalupahana A.W., Wijesekera D.P.H., Kottawatta S.A., Jayasekera N.K., Silva-Fletcher A., Jagoda S.S.S.S. (2021). Characterization and Antimicrobial Resistance of Environmental and Clinical *Aeromonas* Species Isolated from Fresh Water Ornamental Fish and Associated Farming Environment in Sri Lanka. Microorganisms.

[25] Sadique A., Neogi S.B., Bashar T., Sultana M., Johura F.-T., Islam S., Hasan N.A., Huq A., Colwell R.R., Alam M. (2021). Dynamics, Diversity, and Virulence of *Aeromonas* spp. in Homestead Pond Water in Coastal Bangladesh. Front. Public Health.

[26] World Health Organization (WHO) (2023). AWaRe Classification of Antibiotics for Evaluation and Monitoring of Use. https://www.who.int/publications/i/item/WHO-MHP-HPS-EML-2023.04.

[27] Heuzenroeder M.W., Wong C.Y. (1999). Distribution of two hemolytic toxin genes in clinical and environmental isolates of *Aeromonas* spp.: Correlation with virulence in a suckling mouse model. FEMS Microbiol. Lett..

[28] Lau T.V., Puah S.M., Tan J.M.A., Merino S., Puthucheary S.D., Chua K.H. (2023). Flagellar motility mediates biofilm formation in *Aeromonas dhakensis*. Microb. Pathog..

[29] Yin W., Wang Y., Liu L., He J. (2019). Biofilms: The Microbial “Protective Clothing” in Extreme Environments. Int. J. Mol. Sci..

[30] Watnick P., Kolter R. (2000). Biofilm, city of microbes. J. Bacteriol..

[31] Karem K.L., Foster J.W., Bej A.K. (1994). Adaptive acid tolerance response (ATR) in *Aeromonas hydrophila*. Microbiology.

[32] Nojoumi S.A., Smith D.G., Rowbury R.J. (1995). Tolerance to acid in pH 5.0-grown organisms of potentially pathogenic gram-negative bacteria. Lett. Appl. Microbiol..

[33] Scoaris D.O., Colacite J., Nakamura C.V., Ueda-Nakamura T., de Abreu Filho B.A., Dias Filho B.P. (2008). Virulence and antibiotic susceptibility of *Aeromonas* spp. isolated from drinking water. Antonie Van Leeuwenhoek.

[34] Wadström T., Ljungh A. (1991). *Aeromonas* and *Plesiomonas* as food- and waterborne pathogens. Int. J. Food Microbiol..

[35] Nascimento M., Rodrigues J., Reis L., Nogueira I., Carvalho P., Brandão J., Duarte A., Jordao L. (2016). Pathogens in ornamental waters: A pilot study. Int. J. Environ. Res. Public Health.

[36] (2022). The European Committee on Antimicrobial Susceptibility Testing Breakpoint Tables for Interpretation of MICs and Zone Diameters. Version 12.0. https://www.eucast.org/clinical_breakpoints.

[37] Bandeira M., Carvalho P.A., Duarte A., Jordao L. (2014). Exploring dangerous connections between *Klebsiella pneumoniae* biofilms and healthcare-associated infections. Pathogens.

[38] Gavín R., Merino S., Altarriba M., Canals R., Shaw J.G., Tomás J.M. (2003). Lateral flagella are required for increased cell adherence, invasion and biofilm formation by *Aeromonas* spp.. FEMS Microbiol. Lett..

[39] Morgado P.I., Jose S., Wanke R.M., Antunes A.M., Cardoso A.S., Jordao L. (2017). Integration of cellular and molecular endpoints to assess the toxicity of polycyclic aromatic hydrocarbons in HepG_2_ cell line. Environ. Toxicol. Chem..

